# Item

**Published:** 1905-07

**Authors:** 


### ITEM.

A study of the treatment of anemia in Porto Rico has been made by a government commission, appointed in February, 1904, and its report of something more than 200 pages, printed in Spanish and English, has been submitted to the governor of that island.

This particular form of anemia was found to be due to the parasite called hookworm or ucinaria. The commission established a tent hospital and subjected the patients to expert and scientific treatment. Iron was given in various forms, but particularly pepto-mangan (Gude) seemed to find favor. Eighteen cases in which this remedy was administered are given in detail and the results were favorable. This may be regarded as a severe test and those interested will find the report of value.

